# Maternal gestational weight gain and objectively measured physical activity among offspring

**DOI:** 10.1371/journal.pone.0180249

**Published:** 2017-06-29

**Authors:** Niko S. Wasenius, Kimberly P. Grattan, Alysha L. J. Harvey, Nick Barrowman, Gary S. Goldfield, Kristi B. Adamo

**Affiliations:** 1 Faculty of Health Sciences, School of Human Kinetics, University of Ottawa, Ottawa, Canada; 2 Folkhalsan Research Center, Helsinki, Finland; 3 Clinical Research Unit, Children’s Hospital of Eastern Ontario Research Institute (CHEO RI), Ottawa, Canada; 4 Healthy Active Living and Obesity (HALO) Research Group, Ottawa, Canada; University of Tennessee Health Science Center, UNITED STATES

## Abstract

**Objective:**

Animal studies have suggested that maternal weight-related factors during pregnancy can program offspring physical activity in a sex-dependent manner. However, there is limited evidence in humans. The purpose of this study was to investigate the association between maternal gestational weight gain (GWG) and offspring total physical activity (TPA) level and to determine whether these associations are moderated by sex of offspring or maternal pre-pregnancy weight status.

**Method:**

We studied 56 boys (mean age = 3.7 years, standard deviation (SD) 0.5) and 57 girls (mean age = 3.5±0.5 years) enrolled in licensed childcare centers. TPA was objectively measured using Actical^®^ accelerometers. Information on pre-pregnancy body mass index (BMI), GWG, and other maternal factors were collected with a maternal health questionnaire. Associations between GWG, as a continuous variable or categorically (inadequate, adequate, and excessive), and offspring TPA were analysed using linear mixed models to take into account the intraclass correlation between the clusters (childcare centers). Models were adjusted for gestational age, accelerometer weartime, socioeconomic status, and pre-pregnancy BMI status.

**Results:**

We found a significant sex interaction (P-value = 0.009). In boys, greater GWG was associated with decreased offspring TPA (β = -3.2 counts⋅1000^−1^/d, 95% confidence intervals (CI) = -6.4–0.02, P-value = 0.049). In girls born to mothers categorized as overweight or obese, the association between the GWG and TPA followed an inverted U-shape curve (β for GWG squared = -0.1 counts⋅1000^−1^/d, 95% CI = (-0.2 –-0.04), P-value = 0.005). In contrast, a U-shaped curve was found in girls born to mothers classified as lean (pre-pregnancy BMI<25 kg/m^2^) (β for GWG squared = 0.7 counts⋅1000^−1^/d, 95% CI = 0.2–1.2, P-value = 0.011). In boys, TPA in offspring was higher among women with inadequate GWG compared to adequate GWG (P-value = 0.0137), whereas no significant differences were found in girls (P-value = 0.107).

**Conclusion:**

Maternal GWG can be an important biological marker of offspring TPA. These findings support the sex-dependent early developmental programming influence of GWG on TPA.

## Introduction

Physical activity (PA) has been linked to numerous health benefits in children [1,2]. Unfortunately, awareness of these health benefits has not translated into sufficient increases in PA, as a substantial proportion of children do not meet the PA and sedentary behavior guidelines [3]. The current Canadian early years movement guidelines for infants, toddlers, and preschool-aged children call for volume and do not specify intensity [4]. Unfortunately, different intervention strategies and approaches aimed at increasing children’s volume of PA have proven to be relatively ineffective [5,6]. Further information is required about factors that regulate children’s PA behaviour to find more effective strategies to increase their PA levels [7,8].

Like many other complex traits, PA originates from a multitude of interactions between the genome, epigenome, environment, as well as the function and structure of different bodily systems [9]. In addition, the notion that early life exposures can alter our tendency to be physically active in later life has gained popularity [9]. According to the developmental origins of health and disease (DOHaD) concept, the *in utero* and early childhood environment can influence the growth and development of an embryo, fetus, and thus child, in essence “programming” their future life trajectory [10].

Much of the research focusing on the developmental programming paradigm has converged on the long-term risk for non-communicable diseases. Substantial evidence from multiple birth cohort studies has shown that factors such as maternal pre-pregnancy weight status, gestational weight gain (GWG), and birth weight, a surrogate marker for the intrauterine environment, are associated with increased risk of developing non-communicable diseases among offspring [11–13]. Some human studies have also investigated the association between birth weight and offspring self-reported [14] or objectively measured PA later in life [15]. In fact, a meta-analysis of data from 13 birth cohorts found that both low and high birth weight was associated with decreased PA in adult offspring compared to the normal birth weight category [14]. However, studies applying objective PA measurements have not been able to confirm an association between birth weight and PA [15–17].

Although the association between birth weight and PA has not been well established, animal studies have linked a maternal high-fat diet during pregnancy with a reduction in offspring PA [18,19], possibly in a sex-dependent manner [20]. Interestingly, in humans, maternal fat intake during pregnancy may be more strongly related to GWG than to birth weight [20]. GWG has also shown to be independently associated with elevated levels of leptin in fetal cord blood [21]. Subsequently, leptin has been linked to the developmental programming of energy balance regulation [22] and regulation of PA [8]. While the link between GWG and offspring risk for obesity in childhood [23], asthma [24], and several cardio-metabolic risk factors [25] has been established, less is known about whether it is associated with health-related behaviors. Thus, we hypothesized that in our current obesogenic environment, where up to 60% of women exceed current evidence-based guidelines for GWG [26] and children are largely physically inactive [27], GWG could represent a potential modifiable biological candidate marker for children’s PA.

The purpose of this study was to investigate the relationship between maternal GWG and objectively measured daily total PA (TPA) in preschool-aged boys and girls. We also aimed to evaluate whether this association is moderated by sex of offspring or maternal pre-pregnancy weight status.

## Methods

### Subjects

This study uses data collected from the Activity Begins in Childhood (ABC, ISRCTN Registry, ISRCTN94022291) trial [28]. The ABC-trial was a cluster randomized controlled trial that investigated the effect of a 6-month childcare PA intervention with or without a home component on preschoolers’ TPA. The participants were recruited from 18 childcare centers located in Ottawa (Ontario, Canada) and surrounding area. In the present study, we utilized the baseline PA data of preschoolers baseline PA data thus eliminating any potential intervention effects. Our final analyses included 113 singleton children (56 boys and 57 girls). One mother/child dyad was not included due to abnormal maternal GWG (-15 kg) relative to the pre-pregnancy weight (65 kg). The Children’s Hospital of Eastern Ontario Research Ethics Board approved this study and parents provided written informed consent before participation.

### Measurements

#### Total physical activity

TPA was measured for a 7-day period with omni directional Actical® accelerometers (Phillips–Respironics, Ore., USA). Study staff secured the Acticals onto an elasticized belt with frontal clip closure and taught parents and childcare teachers how to correctly position the Actical upright and over the right hip of the children. They were also instructed to remove the Actical during bathing or swimming and to record on a log sheet when the child put on and took off their Actical each day. Trained educators provided the accelerometers to the children at their arrival to the childcare center on day 1 and study staff collected these at the end of the measurement period. Data were collected in 15-second epochs during the waking hours. Continuous zero counts for longer than 60 minutes were considered non-weartime. Days were considered valid if children had at least four hours of accelerometer weartime during childcare hours (from 8:30 am to 4:30 pm) [29] and one hour of weartime during hours outside of childcare (weekdays) or at least 5 hours of weartime during a weekend day. Children were included in the analysis if they had at least 5 hours of accelerometer data per day for at least 3 of the 7-day measurement period [3]. The variability in accelerometer counts and weartime for each valid day is described in Table 1. TPA was expressed as a sum of total daily counts during valid days divided by the number of valid days and reported as counts per day. Accelerometer data was analyzed with a combination of a specific data analysis support tool (SAS, ACCEL+ version 1.0) that has been previously used in the Canadian Health Measures Surveys [30] and a customized Stata program.

**Table 1:** The variability in accelerometer counts and weartime for each valid day.

Day	n (%)		Counts⋅1000^−1^		Weartime	
**Boys**						
**1**	56	(100)	192.8	(68.8)	683.1	(99.6)
**2**	56	(100)	230.9	(98.1)	762.0	(162.4)
**3**	56	(100)	219.1	(87.8)	741.2	(138.4)
**4**	55	(98)	216.1	(87.1)	753.7	(141.9)
**5**	52	(93)	205.2	(85.8)	759.6	(167.5)
**6**	39	(70)	230.9	(113.8)	795.3	(198.1)
**7**	16	(29)	193.1	(97.9)	720.5	(203.7)
**Girls**						
**1**	57	(100)	156.5	(62.4)	643.6	(134.4)
**2**	57	(100)	204.2	(67.6)	727.9	(154.2)
**3**	57	(100)	191.9	(98.2)	671.5	(183.3)
**4**	54	(95)	182.4	(80.3)	668.0	(135.3)
**5**	52	(91)	192.2	(72.3)	719.4	(160.2)
**6**	44	(77)	197.3	(79.2)	677.9	(159.1)
**7**	21	(37)	179.5	(67.0)	696.8	(182.5)

Data are shown as mean (standard deviation) unless otherwise stated

#### Children’s anthropometrics

Child height was measured with a portable stadiometer (Seca GmBH & Co Kg, Hamburg Germany). Weight was measured with a calibrated portable digital scale (ProFit Precision Personal Health Scale, UC-321, A&D Medical, San Jose, CA) to the nearest 0.1 kg. Both height and weight were measured using the standard Canadian Society for Exercise Physiology–Physical Activity Training for Health (CSEP-PATH) protocol [31]. The scale and stadiometer were placed on a clean flat surface. The stadiometer was set-up according to manufacturer’s guidelines that specified using the extension arm to brace against a wall to ensure the stadiometer was entirely upright. The CSEP-PATH protocol specifies that shoes are to be removed and light clothing worn. Each measure was taken twice, and if the measures were within two decimal places of the initial measure, the average was recorded. If any discrepancy beyond two decimal places existed, the participant was re-measured. Child body mass index (BMI) was calculated by dividing weight in kilograms by height in meters squared. Information about child birth weight was based self-report of the mother.

#### Maternal factors

Mothers’ self-reported GWG was measured with a questionnaire. In addition to continuous GWG, we categorized GWG into inadequate, adequate, and excessive according to the Institute of Medicine IOM guidelines [32]. A questionnaire was also administered to collect information about pre-pregnancy height and weight, pregnancy complications, current height and weight, and gestational age at term. The retrospective self-report of pre-pregnancy weight, GWG, and other pregnancy-related factors have been fairly reliable and valid [33–35]. Maternal pre-pregnancy BMI was calculated by dividing pre-pregnancy weight in kilograms by height in metres squared.

#### Socioeconomic status

Socioeconomic status (SES) was defined by household income which was measured with a questionnaire modified from the one used in the Canadian Health Measures Survey and divided income into three categories ($0–29 999, $30 000–99 999, ≥ $100 000) [36].

#### Statistics

Data are reported as mean and standard deviations (SD) or 95% confidence intervals (CI) unless otherwise stated. Baseline characteristics between the boys and girls were compared with a chi-squared test (nominal), Mann-Whitney U test (ordinal), or a t-test (continuous). To account for the clustered design, we used a linear mixed effect model with a random effect for childcare center to investigate the association between the GWG and offspring TPA. A significant sex x GWG interaction effect on TPA was found (P = 0.009). Thus analyses were performed separately for boys and girls. Furthermore, we performed sex-specific analyses stratified by maternal pre-pregnancy BMI. Pre-pregnancy BMI was grouped into only two categories, as we had a limited number cases of underweight or obese mothers. For the first category we pooled underweight and normal weight categories (0 = maternal pre-pregnancy BMI < 25.0 kg/m^2^), and for the second category, we pooled overweight and obese weight categories (1 = maternal pre-pregnancy BMI ≥ 25.0 kg/m^2^). In addition to linear models, we investigated the non-linear quadratic models by including a quadratic term (GWG^2^ = GWG times GWG) into the model, and significant models with higher-order terms were reported. Similar linear mixed effects models were also used to investigate the association between inadequate, adequate, and excessive GWG and offspring TPA based on the aforementioned GWG guidelines [32]. In the case of violation of assumptions (e.g. non-normality), the generalized robust sandwich estimators were used to calculate the standard errors. All models were adjusted for accelerometer weartime, gestational age, and SES. Additionally, when applicable, models were further adjusted for maternal pre-pregnancy BMI status. Analyses were not adjusted for birth weight as it was not significantly associated with TPA in boys (P = 0.219) or girls (P = 0.957). Data were considered statistically significant when p-values were < 0.05. All analyses were performed with Stata 13.1 SE (StataCorp LP, TX, USA).

## Results

Baseline characteristics of participants are presented in Table 2. As shown in Table 2, boys were significantly older, taller, heavier, and were more active than girls. No significant differences were found between sexes in maternal GWG, although mothers with male offspring were more likely to exceed GWG guidelines. We found no significant sex differences in child’s current BMI, maternal pre-pregnancy weight, gestational age, pregnancy complications, or SES. There were no significant differences in age, gestational age, birth weight, body height, body weight, BMI, TPA, or accelerometer weartime between boys and girls included in the analysis (n = 113) and those who were excluded (n = 54) (P > 0.05). Children who were excluded were those who had PA data but were missing data for other variables. SES status, which was only available from 28 (52%) of the excluded children, was significantly higher among included children (χ^2^ = 11.6, df = 1, P = 0.001).

**Table 2:** Subject characteristics.

	Boys			Girls			
Characteristics	n	Mean (SD)		n	Mean (SD)		P-value
**Children**							
** Age (years)**	55	3.7	(0.5)	57	3.5	(0.5)	0.031
** Gestational age (weeks)**	56	39.0	(2.0)	57	38.9	(1.9)	0.951
** Birth weight (g)**	53	3556	(689)	57	3240	(554)	0.009
** Height (cm)**	55	101.7	(6)	57	97.7	(5.3)	<0.001
** Weight (kg)**	55	17.0	(2.5)	57	15.4	(1.9)	<0.001
**BMI (kg/m**^**2**^**)**	55	16.4	(1.3)	57	16.1	(1.4)	0.326
**TPA (counts·1000**^**−1**^**/d)**	56	213.6	(69.6)	57	183.8	(48.9)	0.010
** Weartime (min/d)**	56	742.1	(106.2)	57	679.3	(104.7)	0.002
**Mothers**							
** Pre-pregnancy weight (kg)**	56	65.2	(10.2)	57	67.9	(13.5)	0.234
**Pre-pregnancy BMI (kg/m**^**2**^**)**	56	24.0	(3.8)	57	24.6	(4.6)	0.460
** Pre-pregnancy BMI categories**							0.202
** Underweight, n (%)**	56	0	(0)	57	2	(4)	
** Normal weight, n (%)**	56	42	(75)	57	33	(58)	
** Overweight, n (%)**	56	8	(14)	57	11	(19)	
** Obese, n (%)**	56	6	(11)	57	11	(19)	
** GWG (kg)**	56	13.9	(5.7)	57	12.7	(5.6)	0.302
** GWG categories**							0.011
** Inadequate, n (%)**	56	12	(21)	57	23	(40)	
** Adequate, n (%)**	56	26	(46)	57	25	(44)	
** Excessive, n (%)**	56	18	(32)	57	9	(16)	
** Pregnancy complications**							
** Pre-term delivery, n (%)**	56	3	(5)	57	4	(7)	1.000
** IUGR, n (%)**	56	0	(0)	57	3	(5)	0.243
** GDM, n (%)**	56	2	(4)	57	4	(7)	0.679
** PIH, n (%)**	56	1	(2)	57	3	(5)	0.618
** Other, n (%)**	56	17	(30)	57	18	(32)	1.000
** Household income**							0.465
** ≤ $29,999, n (%)**	56	2	(4)	57	5	(9)	
** $30,000 –$99,999, n (%)**	56	9	(16)	57	9	(16)	
** ≥$100,000, n (%)**	56	45	(80)	57	43	(75)	

BMI, body mass index; TPA, total physical activity; GWG, gestational weight gain; IUGR, intrauterine growth restriction; GDM, gestational diabetes mellitus; PIH, pregnancy induced hypertension

**Fig 1:**
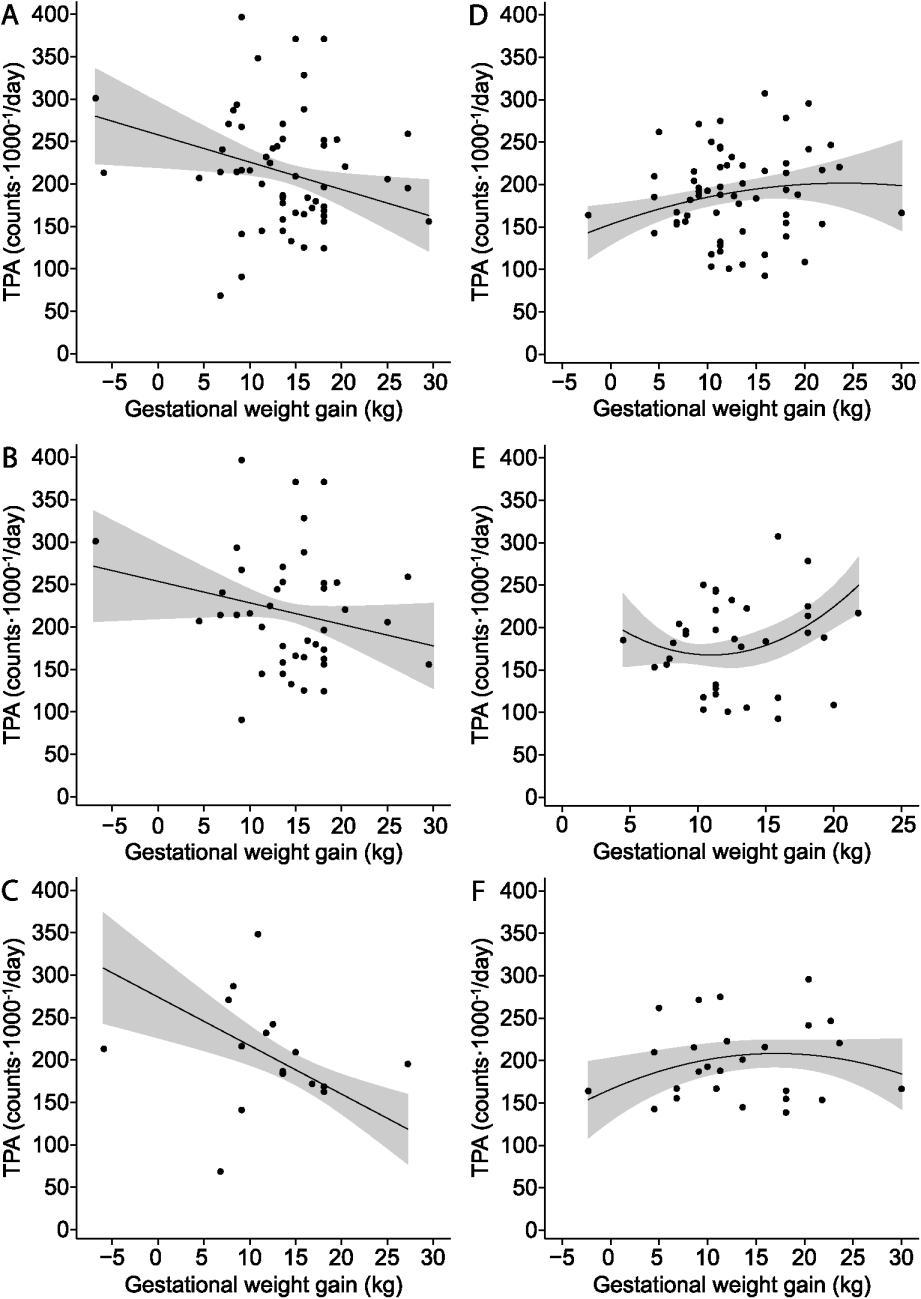
The relationship between the maternal gestational weight gain (GWG) and physical activity in offspring. (A) In all preschool-age boys (n = 56). (B) In preschool age boys born to mothers with pre-pregnancy body mass index (BMI) < 25 kg/m^2^ (n = 42) (C) In preschool age boys born to mothers with pre-pregnancy BMI ≥ 25 kg/m^2^ (n = 14) (D) In all preschool-age girls (n = 57). (E) In preschool-age girls born to mothers with pre-pregnancy BMI < 25 kg/m^2^ (n = 35). (F) In preschool-age girls born to mothers with pre-pregnancy BMI ≥ 25 kg/m^2^ (n = 22). Adjusted for maternal pre-pregnancy BMI (only all), gestational age at term, accelerometer weartime, andeconomic status.

A greater maternal GWG was significantly and linearly associated with decreased TPA in all boys when adjusted for maternal pre-pregnancy BMI, gestational age at term, accelerometer weartime, and SES (Table 3, Fig 1A). We found no significant GWG by maternal pre-pregnancy BMI interaction on male offspring TPA (P for interaction = 0.561). The association between GWG and TPA was similar regardless whether boys were born to mothers who were normal weight or overweight/obese (Table 3, Fig 1B and 1C).

**Table 3:** The relationship between the maternal gestational weight gain and preschool-age male offspring TPA (counts⋅1000^−1^/d) stratified by maternal pre-pregnancy body mass index.

Maternal pre-pregnancy BMI	Independent variable	β (95% CI)		P
All women	GWG	-3.2	(-6.4 –-0.02)	0.049
Normal (BMI <25 kg/m^2^)	GWG	-2.5	(-5.6–0.5)	0.105
Overweight or obese (≥25 kg/m^2^)	GWG	-8.9	(-20.8–3.1)	0.148

BMI, body mass index; GWG, gestational weight gain

For all women n = 56, n for normal n = 42, and for overweight or obese n = 14

All models are adjusted for maternal pre-pregnancy BMI status (only all), gestational age at term, accelerometer weartime, and socioeconomic status.

In analyses including all girls, maternal GWG was not significantly (neither linearly or non-linearly) associated with TPA in their offspring (Table 4, Fig 1D). However, in girls, we found a significant GWG squared by maternal pre-pregnancy BMI interaction on offspring TPA (P for interaction = 0.016). When analyses were stratified by maternal pre-pregnancy BMI, GWG was significantly and non-linearly associated with offspring TPA both in girls born to mothers who were lean or overweight/obese (Table 4). Among girls born to lean mothers, the relationship between GWG and offspring TPA followed a U-shaped curve (Fig 1E). GWG at the vertex, the lowest point of a quadratic curve, was 11 kg and TPA was 167.6 counts⋅1000^−1^/d. In contrast, among girls born to mothers who were overweight or obese the association between GWG and TPA followed a significant inverted U-shaped curve (Fig 1F). At the vertex, the highest point of a quadratic curve, GWG was 18 kg, and TPA was 197.9 counts⋅1000^−1^/d.

**Table 4:** The relationship between the maternal gestational weight gain and TPA in preschool-age female offspring (counts⋅1000^−1^/d) when stratified by maternal pre-pregnancy body mass index.

Maternal pre-pregnancy BMI	independent variable	β (95% CI)		P
All women	GWG	1.7	(-0.6–4.0)	0.147
Normal weight (BMI <25 kg/m^2^)	GWG squared	0.7	(0.2–1.2)	0.011
Overweight or obese (≥25 kg/m^2^)	GWG squared	-0.1	(-0.2–0.04)	0.005

BMI, body mass index; GWG, gestational weight gain

For all women n = 57, for normal weight n = 35, and for overweight or obese n = 22

All models are adjusted for maternal pre-pregnancy BMI status (only all), gestational age at term, accelerometer weartime, and socioeconomic status.

**Fig 2:**
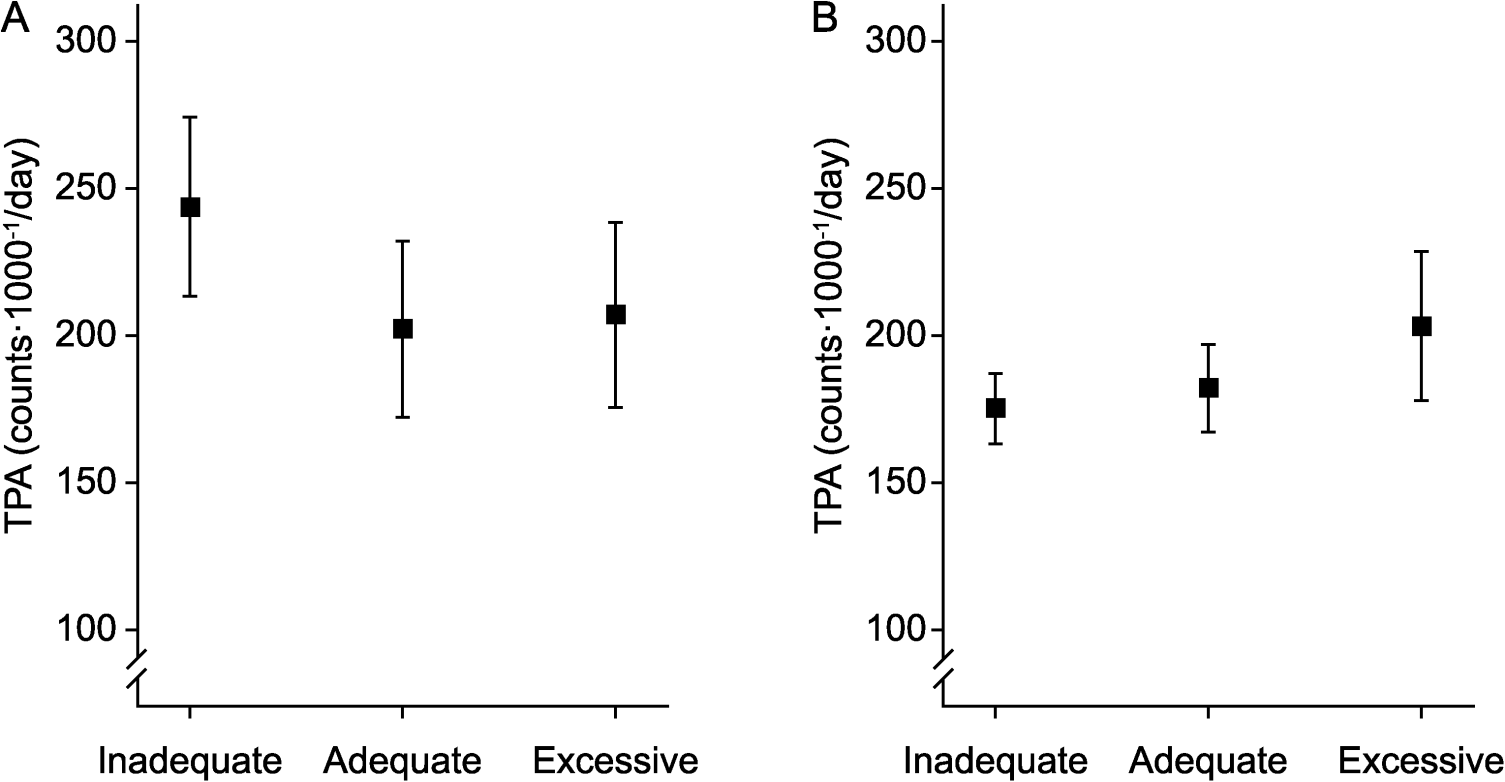
The association between the inadequate, adequate, and excessive gestational weight gain categories and TPA in offspring. (A) Preschool-age boys (n = 56). (B) Preschool-age girls (n = 57).

TPA was significantly different between the inadequate, adequate, and excessive GWG categories in boys when adjusted for gestational age at term, accelerometer weartime, and SES (Fig 2A). In boys, TPA was higher in the inadequate GWG category compared to the adequate (mean difference = -41.6 counts⋅1000^−1^/d, 95% CI = -74.6–8.5, P-value = 0.014) or excessive GWG (mean difference = -36.7 counts⋅1000^−1^/d, 95% CI = -90.6–17.1, P-value = 0.181) categories. No significant differences were found in girls (Fig 2B).

## Discussion

In this study, we found that maternal GWG was independently associated with preschool-age offspring TPA in a sex-dependent manner. In boys, greater GWG was associated with decreased TPA independent of maternal pre-pregnancy BMI status. However, in girls, maternal pre-pregnancy BMI was found to moderate the relationship between GWG and TPA. In mothers who were overweight or obese, the association between the GWG and TPA followed an inverted U-shaped curve, which reached its highest point when GWG was equal to 18 kg. In contrast, a U-shaped curve, which peaked when GWG was equal to 11 kg, was found in mothers who were underweight or normal weight before pregnancy. Additionally, in boys, TPA was found to be higher among the offspring of mothers with inadequate GWG. In girls, TPA did not differ between inadequate, adequate, excessive GWG categories. Findings from the present study support the sex-dependent role of maternal GWG as a predictor of offspring TPA.

We are unaware of any previous human studies that have investigated the relationship between maternal GWG and objectively measured PA in offspring. There are however several animal studies that have linked experimental maternal under- or overnutrition to the developmental programming of PA or locomotor behavior [18,37,38]. In humans, GWG, which has been associated with increased energy, fat and carbohydrate intake [21,39], can be used as a surrogate marker of nutritional status during pregnancy. Although the direct comparison of animal and human studies is difficult, our findings seem consistent with a previous animal study reporting reduced PA in male offspring exposed to the maternal over-nutrition induced by high-fat diet during pregnancy [18]. Furthermore, in female rodents, both a high lard diet [40] and maternal obesity during pregnancy combined with fetal growth restriction [41], have been associated with decreased PA. These results support the inverted U-shaped association found in girls born to overweight or obese mothers (Fig 1F). However, it does not support the U-shaped association found in girls born to lean mothers (Fig 1E). Unfortunately, we were unable to investigate the factors that could explain these differences, and we suspect they could be related to the maternal diet or PA during pregnancy. Previous studies have reported a better diet quality [42–44] and greater volume of PA [45] during pregnancy among lean women compared to the overweight or obese women. Further studies are required to examine, whether there is a specific maternal micro- or macronutrient or PA behavior that could help explain the relationships reported.

In the present study, we also compared the differences in offspring TPA between the inadequate, adequate, and excessive GWG categories as recommended by IOM [32]. In boys, inadequate GWG was associated with higher TPA in offspring, which is consistent with the results from continuous GWG. We did not find significant differences in girls, which could be related to the fact this association may be modified by pre-pregnancy BMI. Unfortunately, we had too few cases to stratify these analyses by maternal pre-pregnancy weight status. Overall, because of the limited sample size, our finding related to GWG categories should be interpreted with caution. In the future, larger studies with objectively measured PA data are required to verify these initial but novel findings. Nevertheless, our current data support maternal GWG as a possible biomarker of sex-specific programming of PA in offspring.

According to a recent review article, the exact mechanisms for sex differences in developmental programming remain to be determined. Then again, these differences may be related to differences in placenta function [46]. Work by our group has recently demonstrated differential expression of placental nutrient transporters in male offspring of women who are obese vs. lean and those who exceed GWG guidelines [47]. Also, maternal obesity has been linked to the sex-specific methylation of the placenta *LEP* gene, which has also been positively associated with neurobehavioral profiles marked by lethargy, hypotonicity, non-optimal reflexes, and low excitability in males [48]. Collectively, the literature demonstrates that there is considerable sexual dimorphism in placenta, that placentas from male and female offspring behave differently and likely alter the nutrient and hormonal *milieu* passed to the fetus. Although the exact mechanisms behind the developmental programming of PA remain to be determined, it could be related to the adverse development of neuronal circuitry in the hypothalamus induced by altered circulating levels of hormones, e.g. insulin or leptin [49]. In animal studies, maternal obesity and overfeeding have been directly linked to hypothalamic leptin resistance, reduced leptin signalling, and altered hypothalamic neurodevelopment towards orexigenic pathways [50], each having been associated with regulation of PA [51]. Hypothalamic leptin resistance could also affect an individual’s PA by down-regulating nescient helix-loop-helix 2 (*Nhlh2*) transcription factor [52]. Nhlh2 has been associated with increased spontaneous PA and voluntary exercise in mice possibly by increasing one’s motivation to exercise by up-regulating b-endorphin and/or dopamine levels [53]. Although such mechanisms cannot be directly investigated in humans, there is evidence showing that maternal obesity during pregnancy and GWG are associated with fetal hyperleptinemia and insulin resistance [21,54] and that high leptin levels at birth are linked to the head circumference, supporting the role of leptin in brain development [55]. In addition to the morphological changes of the brain, suboptimal maternal diet could affect PA indirectly by reducing skeletal muscle mass or strength [56]. It is also likely that these mechanisms interact highly with epigenetic processes [9].

Our findings support the developmental programming of children’s TPA. It is easy to postulate that one of the possible influences that optimizing GWG may have on offspring TPA, and subsequently on children’s health, is related to childhood obesity. In the dataset used in the present study, offspring TPA was not linked to children’s BMI Z score (β = -0.00005 counts⋅1000^−1^/d, 95% CI = -0.003–0.003) when adjusted for accelerometer weartime and SES. While the relationship between TPA and BMI, or obesity prevention, has been shown quite conclusively in older children, at the pre-school age this has been less consistent [57]. Similar to Carson et al. [57] we were unable to identify this relationship in our pre-school aged children, but if the current PA patterns were to continue over time, it is possible that these children would be at risk of obesity. In the future, larger prospective cohort studies are required to investigate a possible influence of GWG related programming of TPA on BMI and other health indicators in offspring.

A notable strength of our study is that it focuses on humans, a rarity when studying fetal programming of PA. An additional strength is the use of objectively measured TPA of at least five hours per day over 3 or more days, which has previously shown to be representative of preschoolers’ daily PA [3]. There are limitations that should be considered when interpreting the findings. Maternal weight-related factors were based on maternal recall, which is susceptible to bias. That said, pregnancy represents a very memorable period of time within a woman’s life, and there are data to suggest that women recall GWG quite reliably between 4–12 years post-pregnancy (r = 0.63) [35]. Recognizing the impact of potential misclassification, efforts have been made by McClure et al. [35] to develop a regression equation to correct estimates of GWG based on retrospective self-report data. Using this technique, the correction of recalled GWG did not alter the main outcomes in our study (Fig 1). Furthermore, our findings in boys may have been affected by a limited number of cases at each end of the GWG distribution. Thus, these data should be interpreted with caution and require replication with a larger dataset. Also, our data were limited to the maternal weight-related factors during pregnancy. Consequently, we were unable to investigate the role or maternal PA, dietary intake, or other behavior-related data from mothers before, during, or after pregnancy, which may have altered our findings [58,59]. In the future, the role of maternal diet, PA, and GWG on different stages of pregnancy needs to be studied as they may be related to offspring PA. Lastly, our study sample included children who attended licensed childcare centers. Use of this captive group may have resulted in a sample that is more focused on families with higher SES (Table 2). In Ontario, Canada, childcare centers are relatively costly. Not only that, but the full-time positions are generally filled with children whose parents both work, thus potentially having a higher household income. Replicating these findings with children from families with lower SES will be necessary.

## Conclusions

In conclusion, the findings of the present study provide preliminary evidence that maternal GWG is independently associated with TPA in preschool-aged offspring in a sex-dependent manner. Our data suggest that GWG, which has been previously associated with risk of downstream disease in offspring [22–24], could be a risk factor for physical inactivity. Our findings support future intervention research to determine whether managing GWG in hopes of optimizing developmental programming will have a positive impact on offspring PA.

## References

[1] Janssen I, Leblanc AG. Systematic review of the health benefits of physical activity and fitness in school-aged children and youth. Int J Behav Nutr Phys Act. 2010; 7:40-5868-7-40. 10.1186/1479-5868-7-40 PMC2885312 20459784

[2] Guinhouya BC, Samouda H, Zitouni D, Vilhelm C, Hubert H. Evidence of the influence of physical activity on the metabolic syndrome and/or on insulin resistance in pediatric populations: a systematic review. Int J Pediatr Obes. 2011; 6:361–388. doi: 10.3109/17477166.2011.605896 21851163 10.3109/17477166.2011.605896

[3] Colley RC, Garriguet D, Adamo KB, Carson V, Janssen I, Timmons BW, et al Physical activity and sedentary behavior during the early years in Canada: a cross-sectional study. Int J Behav Nutr Phys Act. 2013; 10:54-5868-10-54. 10.1186/1479-5868-10-54 PMC3655822 23642258

[4] Tremblay MS, Leblanc AG, Carson V, Choquette L, Connor Gorber S, Dillman C, et al Canadian Physical Activity Guidelines for the Early Years (aged 0–4 years). Appl Physiol Nutr Metab. 2012; 37:345–369. doi: 10.1139/h2012-018 22448608 10.1139/h2012-018

[5] van Sluijs EM, McMinn AM, Griffin SJ. Effectiveness of interventions to promote physical activity in children and adolescents: systematic review of controlled trials. BMJ. 2007; 335:703 doi: 10.1136/bmj.39320.843947.BE 17884863 10.1136/bmj.39320.843947.BE PMC2001088

[6] Metcalf B, Henley W, Wilkin T. Effectiveness of intervention on physical activity of children: systematic review and meta-analysis of controlled trials with objectively measured outcomes (EarlyBird 54). BMJ. 2012; 345:e5888 doi: 10.1136/bmj.e5888 23044984 10.1136/bmj.e5888

[7] Craggs C, Corder K, van Sluijs EM, Griffin SJ. Determinants of change in physical activity in children and adolescents: a systematic review. Am J Prev Med. 2011; 40:645–658. doi: 10.1016/j.amepre.2011.02.025 21565658 10.1016/j.amepre.2011.02.025 PMC3100507

[8] Garland T Jr, Schutz H, Chappell MA, Keeney BK, Meek TH, Copes LE, et al The biological control of voluntary exercise, spontaneous physical activity and daily energy expenditure in relation to obesity: human and rodent perspectives. J Exp Biol. 2011; 214:206–229. doi: 10.1242/jeb.048397 21177942 10.1242/jeb.048397 PMC3008631

[9] Garland T Jr, Cadney MD, Waterland RA. Early-Life Effects on Adult Physical Activity: Concepts, Relevance, and Experimental Approaches. Physiol Biochem Zool. 2017; 90:1–14. doi: 10.1086/689775 28051947 10.1086/689775 PMC6397655

[10] Barker DJ. The origins of the developmental origins theory. J Intern Med. 2007; 261:412–417. doi: 10.1111/j.1365-2796.2007.01809.x 17444880 10.1111/j.1365-2796.2007.01809.x

[11] Whincup PH, Kaye SJ, Owen CG, Huxley R, Cook DG, Anazawa S, et al Birth weight and risk of type 2 diabetes: a systematic review. JAMA. 2008; 300:2886–2897. doi: 10.1001/jama.2008.886 19109117 10.1001/jama.2008.886

[12] Eriksson JG, Sandboge S, Salonen MK, Kajantie E, Osmond C. Long-term consequences of maternal overweight in pregnancy on offspring later health: findings from the Helsinki Birth Cohort Study. Ann Med. 2014; 46:434–438. doi: 10.3109/07853890.2014.919728 24910160 10.3109/07853890.2014.919728

[13] Woo Baidal JA, Locks LM, Cheng ER, Blake-Lamb TL, Perkins ME, Taveras EM. Risk Factors for Childhood Obesity in the First 1,000 Days: A Systematic Review. Am J Prev Med. 2016; 50:761–779. doi: 10.1016/j.amepre.2015.11.012 26916261 10.1016/j.amepre.2015.11.012

[14] Andersen LG, Angquist L, Gamborg M, Byberg L, Bengtsson C, Canoy D, et al Birth weight in relation to leisure time physical activity in adolescence and adulthood: meta-analysis of results from 13 nordic cohorts. PLoS One. 2009; 4:e8192 doi: 10.1371/journal.pone.0008192 20016780 10.1371/journal.pone.0008192 PMC2790716

[15] Ridgway CL, Brage S, Sharp SJ, Corder K, Westgate KL, van Sluijs EM, et al Does birth weight influence physical activity in youth? A combined analysis of four studies using objectively measured physical activity. PLoS One. 2011; 6:e16125 doi: 10.1371/journal.pone.0016125 21264270 10.1371/journal.pone.0016125 PMC3020226

[16] Kehoe SH, Krishnaveni GV, Veena SR, Hill JC, Osmond C, Kiran, et al Birth size and physical activity in a cohort of Indian children aged 6–10 years. J Dev Orig Health Dis. 2012; 3:245–252. doi: 10.1017/S2040174412000189 24098836 10.1017/S2040174412000189 PMC3790308

[17] Tikanmäki M, Tammelin T, Kaseva N, Sipola-Leppänen M, Matinolli HM, Hakonen H, et al Objectively measured physical activity and sedentary time in young adults born preterm-The ESTER study. Pediatr Res. 2017. 10.1038/pr.2016.262 27935902

[18] Cunha Fda S, Dalle Molle R, Portella AK, Benetti Cda S, Noschang C, Goldani MZ, et al Both food restriction and high-fat diet during gestation induce low birth weight and altered physical activity in adult rat offspring: the "Similarities in the Inequalities" model. PLoS One. 2015; 10:e0118586 doi: 10.1371/journal.pone.0118586 25738800 10.1371/journal.pone.0118586 PMC4349804

[19] Johnson SA, Javurek AB, Painter MS, Murphy CR, Conard CM, Gant KL, et al Effects of a maternal high-fat diet on offspring behavioral and metabolic parameters in a rodent model. J Dev Orig Health Dis. 2017; 8:75–88. doi: 10.1017/S2040174416000490 27609493 10.1017/S2040174416000490

[20] Lagiou P, Tamimi RM, Mucci LA, Adami HO, Hsieh CC, Trichopoulos D. Diet during pregnancy in relation to maternal weight gain and birth size. Eur J Clin Nutr. 2004; 58:231–237. doi: 10.1038/sj.ejcn.1601771 14749741 10.1038/sj.ejcn.1601771

[21] Logan CA, Bornemann R, Koenig W, Reister F, Walter V, Fantuzzi G, et al Gestational Weight Gain and Fetal-Maternal Adiponectin, Leptin, and CRP: results of two birth cohorts studies. Sci Rep. 2017; 7:41847 doi: 10.1038/srep41847 28150815 10.1038/srep41847 PMC5288774

[22] Breton C. The hypothalamus-adipose axis is a key target of developmental programming by maternal nutritional manipulation. J Endocrinol. 2013; 216:R19–31. doi: 10.1530/JOE-12-0157 23108716 10.1530/JOE-12-0157

[23] Mamun AA, Mannan M, Doi SA. Gestational weight gain in relation to offspring obesity over the life course: a systematic review and bias-adjusted meta-analysis. Obes Rev. 2014; 15:338–347. doi: 10.1111/obr.12132 24321007 10.1111/obr.12132

[24] Forno E, Young OM, Kumar R, Simhan H, Celedon JC. Maternal obesity in pregnancy, gestational weight gain, and risk of childhood asthma. Pediatrics. 2014; 134:e535–46. doi: 10.1542/peds.2014-0439 25049351 10.1542/peds.2014-0439 PMC4187236

[25] Hrolfsdottir L, Rytter D, Olsen SF, Bech BH, Maslova E, Henriksen TB, et al Gestational weight gain in normal weight women and offspring cardio-metabolic risk factors at 20 years of age. Int J Obes (Lond). 2015; 39:671–676. 25298277 10.1038/ijo.2014.179

[26] Ferraro ZM, Barrowman N, Prud'homme D, Walker M, Wen SW, Rodger M, et al Excessive gestational weight gain predicts large for gestational age neonates independent of maternal body mass index. J Matern Fetal Neonatal Med. 2012; 25:538–542. doi: 10.3109/14767058.2011.638953 22081936 10.3109/14767058.2011.638953

[27] Colley RC, Garriguet D, Janssen I, Craig CL, Clarke J, Tremblay MS. Physical activity of Canadian children and youth: accelerometer results from the 2007 to 2009 Canadian Health Measures Survey. Health Rep. 2011; 22:15–23. 21510586

[28] Adamo KB, Barrowman N, Naylor PJ, Yaya S, Harvey A, Grattan KP, et al Activity Begins in Childhood (ABC)—inspiring healthy active behaviour in preschoolers: study protocol for a cluster randomized controlled trial. Trials. 2014; 15:305 doi: 10.1186/1745-6215-15-305 25073797 10.1186/1745-6215-15-305 PMC4124151

[29] Goldfield GS, Harvey AL, Grattan KP, Temple V, Naylor PJ, Alberga AS, et al Effects of Child Care Intervention on Physical Activity and Body Composition. Am J Prev Med. 2016; 51:225–231. doi: 10.1016/j.amepre.2016.03.024 27180030 10.1016/j.amepre.2016.03.024

[30] Colley RC. Actical Accelerometer Data Analysis Support Tool: Harmonizing with the Canadian Health Measures Survey (Accel+). 2012; Available from: www.haloresearch.ca/accel. Accessed 14 January, 2016.

[31] Canadian Society for Exercise Physiology. Canadian Society for Exercise Physiology-Physical Activity Training for Health (CSEP-PATH). Ottawa, ON: Canadian Society for Exercise Physiology; 2013.

[32] IOM. Institute of Medicine (US) and National Research Council (US) Committee to Reexamine IOM Pregnancy Weight Guidelines; 2009.

[33] Tomeo CA, Rich-Edwards JW, Michels KB, Berkey CS, Hunter DJ, Frazier AL, et al Reproducibility and validity of maternal recall of pregnancy-related events. Epidemiology. 1999; 10:774–777. 10535796

[34] Biro FM, Wiley-Kroner B, Whitsett D. Perceived and measured weight changes during adolescent pregnancy. J Pediatr Adolesc Gynecol. 1999; 12:31–32. doi: 10.1016/S1083-3188(00)86618-8 9929838 10.1016/S1083-3188(00)86618-8

[35] McClure CK, Bodnar LM, Ness R, Catov JM. Accuracy of maternal recall of gestational weight gain 4 to 12 years after delivery. Obesity (Silver Spring). 2011; 19:1047–1053. 21164507 10.1038/oby.2010.300 PMC3123900

[36] Tremblay M, Wolfson M, Connor Gorber S. Canadian Health Measures Survey: rationale, background and overview. Health Rep. 2007; 18 Suppl:7–20. 18210866

[37] Johnson SA, Javurek AB, Painter MS, Murphy CR, Conard CM, Gant KL, et al Effects of a maternal high-fat diet on offspring behavioral and metabolic parameters in a rodent model. J Dev Orig Health Dis. 2017; 8:75–88. doi: 10.1017/S2040174416000490 27609493 10.1017/S2040174416000490

[38] Vickers MH, Breier BH, McCarthy D, Gluckman PD. Sedentary behavior during postnatal life is determined by the prenatal environment and exacerbated by postnatal hypercaloric nutrition. Am J Physiol Regul Integr Comp Physiol. 2003; 285:R271–3. doi: 10.1152/ajpregu.00051.2003 12794001 10.1152/ajpregu.00051.2003

[39] Diemert A, Lezius S, Pagenkemper M, Hansen G, Drozdowska A, Hecher K, et al Maternal nutrition, inadequate gestational weight gain and birth weight: results from a prospective birth cohort. BMC Pregnancy Childbirth. 2016; 16:224-016-1012-y. 10.1186/s12884-016-1012-y PMC4986204 27528213

[40] Khan IY, Taylor PD, Dekou V, Seed PT, Lakasing L, Graham D, et al Gender-linked hypertension in offspring of lard-fed pregnant rats. Hypertension. 2003; 41:168–175. 12511548 10.1161/01.hyp.0000047511.97879.fc

[41] Baker MS, Li G, Kohorst JJ, Waterland RA. Fetal growth restriction promotes physical inactivity and obesity in female mice. Int J Obes (Lond). 2015; 39:98–104. 23924758 10.1038/ijo.2013.146 PMC3872504

[42] Shin D, Lee KW, Song WO. Pre-Pregnancy Weight Status Is Associated with Diet Quality and Nutritional Biomarkers during Pregnancy. Nutrients. 2016; 8:162 doi: 10.3390/nu8030162 26978398 10.3390/nu8030162 PMC4808890

[43] Laraia BA, Bodnar LM, Siega-Riz AM. Pregravid body mass index is negatively associated with diet quality during pregnancy. Public Health Nutr. 2007; 10:920–926. doi: 10.1017/S1368980007657991 17381955 10.1017/S1368980007657991

[44] Tsigga M, Filis V, Hatzopoulou K, Kotzamanidis C, Grammatikopoulou MG. Healthy Eating Index during pregnancy according to pre-gravid and gravid weight status. Public Health Nutr. 2011; 14:290–296. doi: 10.1017/S1368980010001989 20642871 10.1017/S1368980010001989

[45] Bacchi E, Bonin C, Zanolin ME, Zambotti F, Livornese D, Dona S, et al Physical Activity Patterns in Normal-Weight and Overweight/Obese Pregnant Women. PLoS One. 2016; 11:e0166254 doi: 10.1371/journal.pone.0166254 27829017 10.1371/journal.pone.0166254 PMC5102361

[46] Clifton VL. Review: Sex and the human placenta: mediating differential strategies of fetal growth and survival. Placenta. 2010; 31 Suppl:S33–9. 20004469 10.1016/j.placenta.2009.11.010

[47] Brett KE, Ferraro ZM, Holcik M, Adamo KB. Placenta nutrient transport-related gene expression: the impact of maternal obesity and excessive gestational weight gain. J Matern Fetal Neonatal Med. 2016; 29:1399–1405. doi: 10.3109/14767058.2015.1049522 26067267 10.3109/14767058.2015.1049522

[48] Lesseur C, Armstrong DA, Murphy MA, Appleton AA, Koestler DC, Paquette AG, et al Sex-specific associations between placental leptin promoter DNA methylation and infant neurobehavior. Psychoneuroendocrinology. 2014; 40:1–9. doi: 10.1016/j.psyneuen.2013.10.012 24485470 10.1016/j.psyneuen.2013.10.012 PMC3912462

[49] Plagemann A. A matter of insulin: developmental programming of body weight regulation. J Matern Fetal Neonatal Med. 2008; 21:143–148. doi: 10.1080/14767050801929869 18297568 10.1080/14767050801929869

[50] Wang H, Ji J, Yu Y, Wei X, Chai S, Liu D, et al Neonatal Overfeeding in Female Mice Predisposes the Development of Obesity in their Male Offspring via Altered Central Leptin Signalling. J Neuroendocrinol. 2015; 27:600–608. doi: 10.1111/jne.12281 25855235 10.1111/jne.12281

[51] Garland T Jr, Schutz H, Chappell MA, Keeney BK, Meek TH, Copes LE, et al The biological control of voluntary exercise, spontaneous physical activity and daily energy expenditure in relation to obesity: human and rodent perspectives. J Exp Biol. 2011; 214:206–229. doi: 10.1242/jeb.048397 21177942 10.1242/jeb.048397 PMC3008631

[52] Al Rayyan N, Zhang J, Burnside AS, Good DJ. Leptin signaling regulates hypothalamic expression of nescient helix-loop-helix 2 (Nhlh2) through signal transducer and activator 3 (Stat3). Mol Cell Endocrinol. 2014; 384:134–142. doi: 10.1016/j.mce.2014.01.017 24486192 10.1016/j.mce.2014.01.017 PMC3984914

[53] Good DJ, Li M, Deater-Deckard K. A Genetic Basis for Motivated Exercise. Exerc Sport Sci Rev. 2015; 43:231–237. doi: 10.1249/JES.0000000000000057 26196864 10.1249/JES.0000000000000057

[54] Catalano PM, Presley L, Minium J, Hauguel-de Mouzon S. Fetuses of obese mothers develop insulin resistance in utero. Diabetes Care. 2009; 32:1076–1080. doi: 10.2337/dc08-2077 19460915 10.2337/dc08-2077 PMC2681036

[55] Gohlke BC, Huber A, Bartmann P, Fimmers R, Hecher K, Bouret SG, et al Cord blood leptin and IGF-I in relation to birth weight differences and head circumference in monozygotic twins. J Pediatr Endocrinol Metab. 2006; 19:3–9. 16509522 10.1515/jpem.2006.19.1.3

[56] Du M, Yan X, Tong JF, Zhao J, Zhu MJ. Maternal obesity, inflammation, and fetal skeletal muscle development. Biol Reprod. 2010; 82:4–12. doi: 10.1095/biolreprod.109.077099 19516021 10.1095/biolreprod.109.077099 PMC2802110

[57] Kuzik N, Carson V. The association between physical activity, sedentary behavior, sleep, and body mass index z-scores in different settings among toddlers and preschoolers. BMC Pediatr. 2016; 16:100 doi: 10.1186/s12887-016-0642-6 27439395 10.1186/s12887-016-0642-6 PMC4972189

[58] Eclarinal JD, Zhu S, Baker MS, Piyarathna DB, Coarfa C, Fiorotto ML, et al Maternal exercise during pregnancy promotes physical activity in adult offspring. FASEB J. 2016; 30:2541–2548. doi: 10.1096/fj.201500018R 27033262 10.1096/fj.201500018R PMC4904289

[59] Mattocks C, Ness A, Deere K, Tilling K, Leary S, Blair SN, et al Early life determinants of physical activity in 11 to 12 year olds: cohort study. BMJ. 2008; 336:26–29. doi: 10.1136/bmj.39385.443565.BE 18037616 10.1136/bmj.39385.443565.BE PMC2174780

