# Correction: Maternal gestational weight gain and objectively measured physical activity among offspring

**DOI:** 10.1371/journal.pone.0192284

**Published:** 2018-01-30

**Authors:** Niko S. Wasenius, Kimberly P. Grattan, Alysha L. J. Harvey, Nick Barrowman, Gary S. Goldfield, Kristi B. Adamo

Fig 1, “The relationship between the maternal gestational weight gain (GWG) and physical activity in offspring,” is incorrect. Please see the corrected Fig 1 here

**Fig 1:**
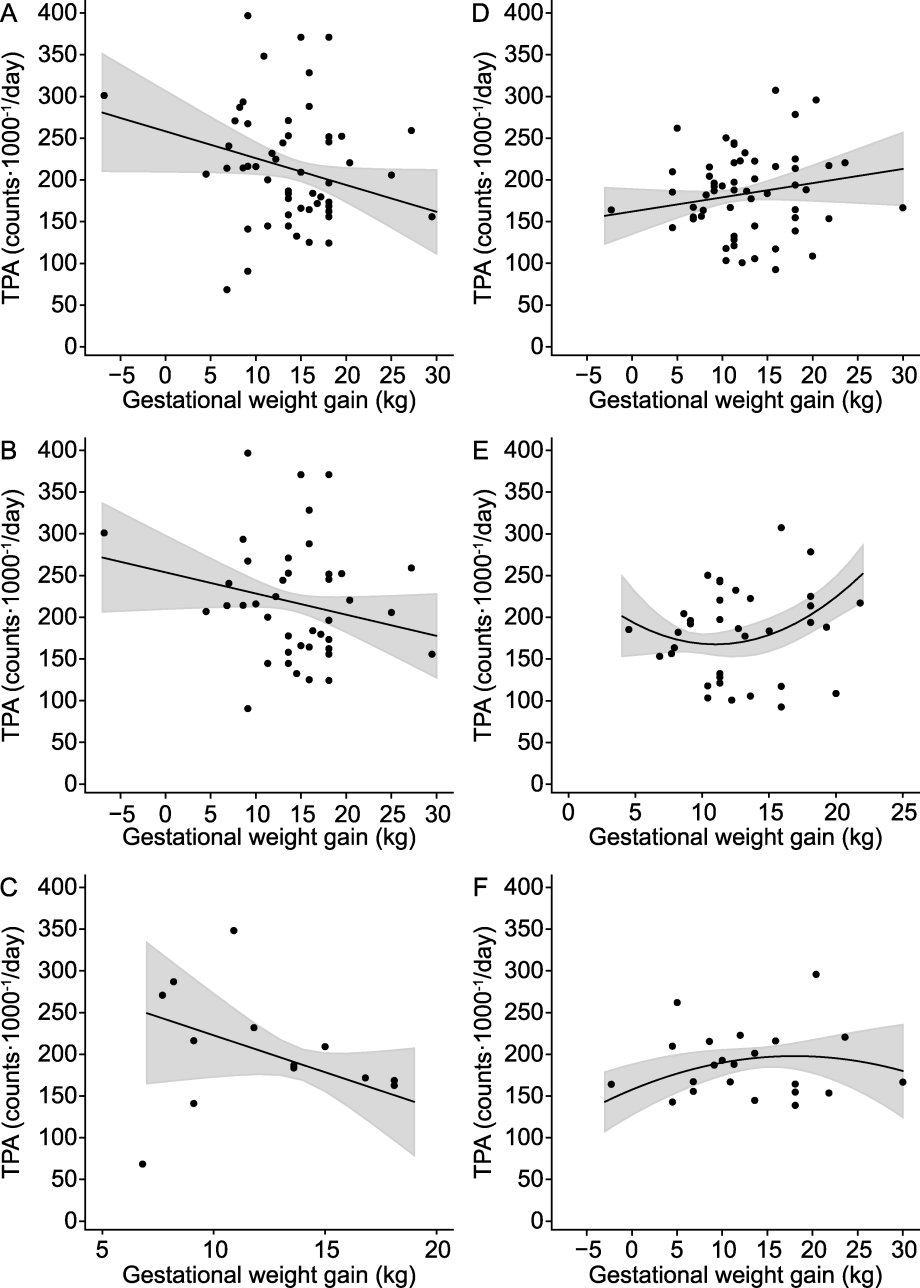
The relationship between the maternal gestational weight gain (GWG) and physical activity in offspring. (A) In all preschool-age boys (n = 56). (B) In preschool age boys born to mothers with pre-pregnancy body mass index (BMI) < 25 kg/m2 (n = 42) (C) In preschool age boys born to mothers with pre-pregnancy BMI ≥ 25 kg/m2 (n = 14) (D) In all preschool-age girls (n = 57). (E) In preschool-age girls born to mothers with pre-pregnancy BMI < 25 kg/m2 (n = 35). (F) In preschool-age girls born to mothers with pre-pregnancy BMI ≥ 25 kg/m2 (n = 22). Adjusted for maternal pre-pregnancy BMI (only all), gestational age at term, accelerometer weartime, andeconomic status.
